# Cutoff Finder: A Comprehensive and Straightforward Web Application Enabling Rapid Biomarker Cutoff Optimization

**DOI:** 10.1371/journal.pone.0051862

**Published:** 2012-12-14

**Authors:** Jan Budczies, Frederick Klauschen, Bruno V. Sinn, Balázs Győrffy, Wolfgang D. Schmitt, Silvia Darb-Esfahani, Carsten Denkert

**Affiliations:** 1 Institut für Pathologie, Charité – Universitätsmedizin Berlin, Berlin, Germany; 2 Research Laboratory of Pediatrics and Nephrology, Hungarian Academy of Sciences, Budapest, Hungary; University Medical Centre Utrecht, The Netherlands

## Abstract

Gene or protein expression data are usually represented by metric or at least ordinal variables. In order to translate a continuous variable into a clinical decision, it is necessary to determine a cutoff point and to stratify patients into two groups each requiring a different kind of treatment. Currently, there is no standard method or standard software for biomarker cutoff determination. Therefore, we developed *Cutoff Finder*, a bundle of optimization and visualization methods for cutoff determination that is accessible online. While one of the methods for cutoff optimization is based solely on the distribution of the marker under investigation, other methods optimize the correlation of the dichotomization with respect to an outcome or survival variable. We illustrate the functionality of *Cutoff Finder* by the analysis of the gene expression of estrogen receptor (ER) and progesterone receptor (PgR) in breast cancer tissues. This distribution of these important markers is analyzed and correlated with immunohistologically determined ER status and distant metastasis free survival. *Cutoff Finder* is expected to fill a relevant gap in the available biometric software repertoire and will enable faster optimization of new diagnostic biomarkers. The tool can be accessed at http://molpath.charite.de/cutoff.

## Introduction

The use of diagnostic, prognostic and predictive biomarkers is of increasing importance in many areas of medicine. Many markers are measured in laboratory assay as continuous variables or as ordinal variables. Gene expression is usually measured on a metric scale, using qRT-PCR or hybridization based methods, for example using single gene assays such as TaqMan or on a global scale using DNA microarrays. In tissue-based diagnostics, protein expression is usually evaluated by immunohistochemistry (IHC) and quantified on an ordinal scale using the percentage of stained cells, staining intensity or combinations of these. Examples of such combinations include the Allred score that takes values between 0 and 8 [1], [2] and the immunoreactive score (IRS) that takes values between 0 and 12 [3], [4].

In order to translate a continuous or ordinal diagnostic variable into a clinical decision, it is necessary to determine a cutoff point and to stratify patients into distinct groups each requiring a different kind of treatment [5]. Although the mean or median value of the diagnostic factor was used before, it is often desirable to determine cutoff points based on the distribution of the variable or by optimizing the correlation with clinical outcome or response to a treatment. Methods for cutoff optimization include minimization of p-values or maximization of combinations of test sensitivity and test specificity [6]. X-Tile is a freely available tool that is specialized to the analysis of survival data and uses a minimal p-value approach for cutoff optimization [7]. Previously, like many others, we manually investigated and optimized cutoff points of molecular markers [8]–[10]. However, methods for cut-off determination vary among published studies and the underlying algorithms remain obscure in many instances.

The literature is filled with newly identified biomarkers, but only a small minority has found acceptance in patient care so far. This is in part due to overestimation of the true effects in the first study and a low reproducibility in subsequent validation studies. Cutoff optimization was demonstrated to contribute to overestimation of results when multiple cutoff points are investigated and the problem of multiple hypothesis testing is ignored [11], [12]. Conflicting the need of a higher degree of objectivity [13], cut-off point determination is often done in a non-systematic manner and therefore among the causes for a poor reproducibility of biomarker studies.

**Figure 1:**
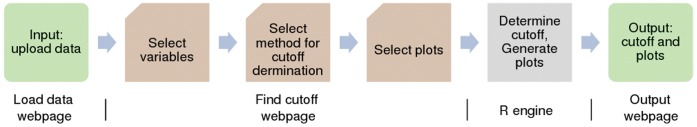
Workflow of Cutoff Finder. The track below the icons refers to the places where the steps of data processing are done. These are the steps of data procession: 1. Data are uploaded from a tab-separated file or imported from one of three example data sets. 2. The user selects the biomarker and optionally outcome and survival variables from the table columns. 3. The user selects the method for cutoff determination. 4. The user chooses the set of plots to be generated. 5. The optimal cutoff point is determined and analysis plots are generated using R as statistical engine. 6. Cutoff point and plots are shown at the results webpage.

The reason for this is the lack of comprehensive and easy-to-use tools for cutoff determination. Therefore, we developed *Cutoff Finder*, a bundle of optimization and visualization methods that are accessible via the internet (http://molpath.charite.de/cutoff). The purpose of the web application is twofold: (i) As not a single methods can be considered to be optimal for all kind of data and all clinical situations, we implemented a multitude of five methods for cutoff determination. (ii) To avoid overestimation of the true effect, the robustness when varying the cutoff point can be investigated in overview plots showing effect sizes with confidence intervals. The workflow through the web application is shown in Fig. 1.

## Methods

### Implementation and Availability

*Cutoff Finder* is a freely available web application that can be accessed using an arbitrary web browser (http://molpath.charite.de/cutoff). The web application is implemented as Java Server Pages (JSPs) that connect to R using the R package and TCP/IP server *Rserve* [14]. The R statistical language [15] serves as an engine for all statistical computing and visualization. The R code for cutoff optimization and the plot functions can be found in Text S1. The R file is loaded as static source to the R server. For each cutoff analysis, the web application calls the wrapper R function *get.cutoff()* that serves as controller. The controller calls specialized R functions for each kind of cutoff optimization and for each kind of plot. Results of the cutoff optimization and plots are returned to the web server. Using the R code, results and plots of the web page can be reproduced 100%.

**Figure 2:**
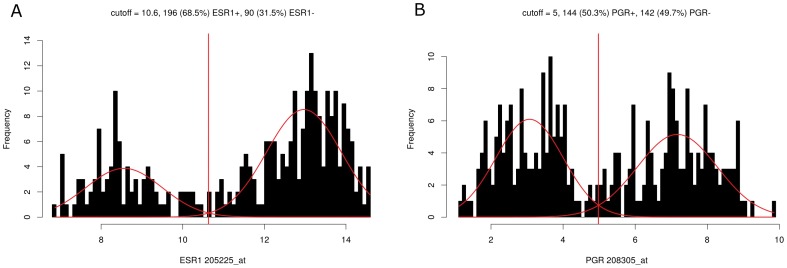
Distribution based cutoff optimization (independent of outcome and survival data) in the GSE2034 breast cancer data. Histograms of ER (**A**) and PgR (**B**) gene expression in 286 lymph-node negative breast cancers. A mixture model of two Gaussian distributions is fitted to each of the histograms (red lines). Vertical lines designate the optimal cutoffs derived from the mixture model.

### Analysis Workflow

The steps of data procession are shown in Figure 1:

1. The data are uploaded from a tab-separated table, rows representing patients and columns representing variables. The maximum number of rows is 5000, the maximum number of columns is 50.
2. The user selects the biomarker and optionally outcome and survival variables from the table columns.
3. The user selects the method for cutoff determination.
4. The user chooses the set of plots to be generated.
5. The inquiry is send to the statistical engine R. The optimal cutoff point is determined and analysis plots are generated.
6. The user is directed to the results webpage, where the optimal cutoff point and plots are shown.

**Figure 3:**
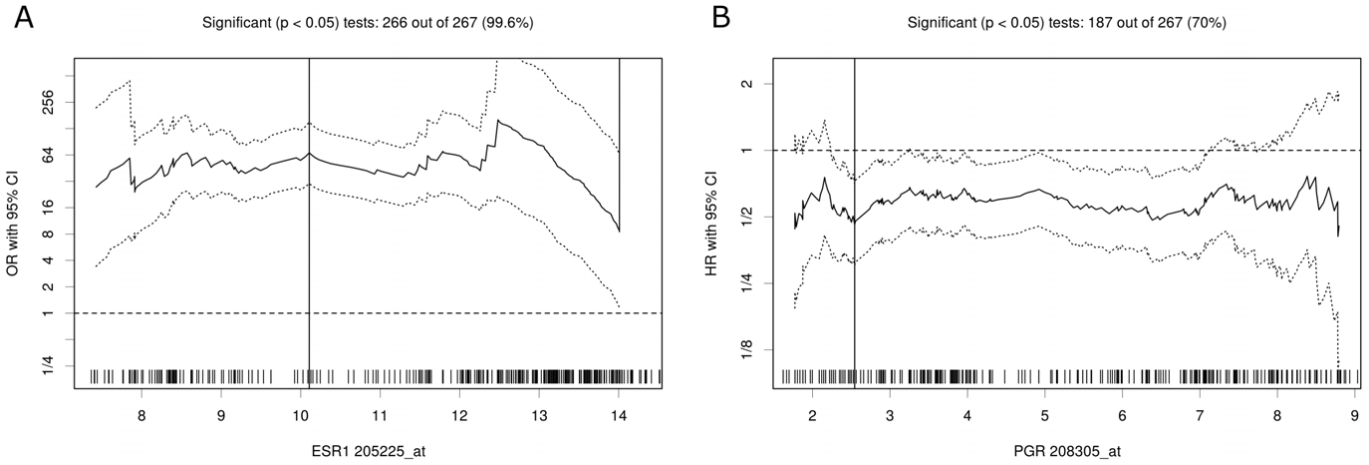
Cutoff optimization by correlation with a binary variable or survival in the GSE2034 breast cancer data. (**A**) For each possible cutoff, ESR1 gene expression is correlated with the immunohistologically determined ER status. The odds ratio (OR) including 95% CI is plotted in dependence of the cutoff. A vertical line designates the dichotomization showing the most significant correlation with immunohistologically determined ER status. (**B**) For each possible cutoff, PgR gene expression was correlated with distance metastasis free survival. The hazard ratio (HR) including 95% CI is plotted in dependence of the cutoff. A vertical line designates the dichotomization showing the most significant correlation with survival. The distribution of the gene expression values in the 286 tumors is shown as a rug plot at the bottom of the figures.

### Cutoff Determination

A multitude of five methods is offered for cutoff optimization. The first method is based solely on the distribution of the biomarker in the sample cohort. Methods 2–4 deal with optimization of the correlation with a binary variable, for example response to treatment. The last method is devoted to optimization of the correlation with survival.

1. Fit of mixture model: A mixture model of two Gaussian distributions is fitted to the histogram of the biomarker. This procedure is implemented using the function *flexmix* from the R package flexmix [16]. The optimal cutoff is determined as the value where the probability density functions of the mixing distribution coincide.
2. Significance of correlation with binary variable: This method correlates the dichotomized biomarker with a binary outcome variable using logistic regression. Logistic regression is executed using the function *glm* from R package stats [15]. The optimal cutoff is defined as the point with the most significant (Fisher’s exact test) split. Odds ratios (ORs) as well as sensitivity and specificity including 95% confidence intervals are calculated. Confidence intervals for proportions are estimated using Wilson’s method as it is implemented in the R package binom [17].
3. Based on ROC curve: Two methods determine the cutoff point by minimizing the distance on the ROC curve to the left top edge of the diagram. The first method minimizes the Euclidean distance between these points. The second method minimizes the Manhattan distance between the points. Here, the sum of sensitivity and specificity is maximized, equivalent to maximization of Youden’s statistics J = sensitivity+specificity –1 [18].
4. Minimum sensitivity or specificity: For each or these two methods, the user enters a percentage value. The cutoff point is chosen as the first threshold for the biomarker, where the sensitivity (or specificity) exceeds this predefined value.
5. Significance of correlation with survival variable: This method fits Cox proportional hazard models to the dichotomized variable and the survival variable. Survival analysis is executed using the functions *coxph* and *survfit* from the R package survival [19]. The optimal cutoff is defined as the point with the most significant (log-rank test) split. Hazard ratios (HRs) including 95% confidence intervals are calculated.

**Figure 4:**
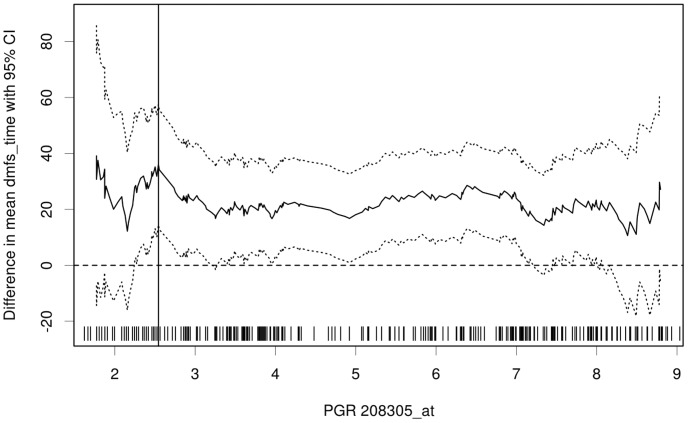
Plot of the differences in survival time. The mean survival time is estimated in the samples where the PgR is highly expressed and in the samples where the PgR is lowly expressed. The difference of the mean survival times including is plotted. The distribution of PgR expression is shown as rug plot at the bottom of the figure.

Additionally, the user can manually enter a cutoff point that is used for calculations and visualization.

**Figure 5:**
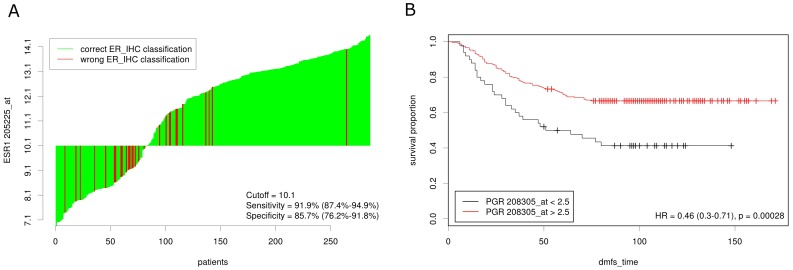
Detailed analysis of the optimal dichotomization of the GSE2034 breast cancer data. (**A**) Comparison of gene expression based and immunohistochemical determination of estrogen receptor status. The classification using ESR1 expression and the optimal cutoff taken from Fig. 2A is compared to the IHC result. (**B**) Kaplan Meier analysis of PgR expression using the optimal cutoff taken from Fig. 2B. Distant metastasis free survival (dmfs) was significantly longer for patients with PgR expression above the cutoff.

### Visualization

Two different kinds of plots can be generated: overview plots and plots at the cutoff point. Overview plots give a summary of all possible cutoff points with the optimal cutoff marked by a vertical line. The second kinds of plots are Waterfall and Kaplan-Meier plots that are generated for a fixed cutoff point. The overview plots include plots of ORs, HRs and differences in survival. ORs are calculated using the function *glm* form the R package stats [15]. HRs are calculated using the function *coxph* from the R package survival [19]. Differences in survival are calculated from the mean survival times in the good prognosis and the poor prognosis group. Mean survival times are estimated from the area under the Kaplan-Meier curve using the maximum time that occurs in the data as uniform time endpoint.

**Table 1:** Cutoff point optimization for estrogene receptor (ER) expression (reporter 205225_at).

Method	GSE2034			GSE7390			GSE11121		
Variable	cutoff	sens.	spec.	cutoff	sens.	spec.	cutoff	sens.	spec.
Fit of mixure model	10.6	88.5%	85.7%	9.6	92.5%	81.2%	11.2	NA	NA
Optimization significance	10.1	91.9%	85.7%	10.4	91.0%	84.4%	NA	NA	NA
Optimization Euclidean distance	10.1	91.9%	85.7%	10.4	91.0%	84.4%	NA	NA	NA
Optimization Manhattan distance	10.1	91.9%	85.7%	10.7	98.6%	84.4%	NA	NA	NA

Comparison of four methods for cutoff optimization. Sensitivity and specificity were calculated by comparison with immunohistochemistry that is the current gold standard.

* no IHC data available for GSE11121.

### Example Data Sets

To illustrate the functionality of *Cutoff Finder* we analyzed gene expression data from breast cancer tissues. To this end, the gene expression series GSE2034, GSE7390 and GSE11121 were downloaded from the Gene Expression Omnibus (GEO) repository [20], [21]. We analyzed measurements of probe 205225_at for estrogen receptor (ESR1) and of 208305_at for progesterone receptor (PgR). The gene expression data are analyzed on log scale, such that a difference of can be interpreted as fold change of 2.

**Table 2:** Cutoff point optimization for progesterone receptor (PgR) expression (reporter 208305_at).

Method	GSE2034			GSE7390			GSE11121		
Variable	cutoff	HR	p	cutoff	HR	p	cutoff	HR	p
Fit of mixure model	5.0	0.65	0.024	4.2	0.53	0.0011	5.6	0.52	0.025
Optimization significance	2.5	0.46	0.0003	5.0	0.47	0.0024	3.7	0.40	0.0018

Comparison of two methods for cutoff optimization. The capability of the cutoffs to stratify into high and a row risk patients was investigated by calculation of hazard ratio and significance of the correlation with distance metastasis free survival.

## Results

We illustrate the functionality of *Cutoff Finder* by the analysis of the gene expression of estrogen receptor (ER) and progesterone receptor (PgR) in breast cancer tissues. In routine diagnostic protocols, ER and PgR status are determined based on the percentage of positive cells in immunohistochemically stained tissue sections. As an alternative approach, assessment of hormone receptor status using gene expression is currently under investigation in several studies [22]. Therefore, we extracted expression data of ER and PgR from the publicly available microarray data sets GSE2034 (286 patients), GSE7390 (198 patients) and GSE11121 (200 patients). Cutoff points were defined for ER and PgR and the correlation of the resulting split with immunohistochemically determined ER status and distance metastasis free survival was analyzed. All the figures presented in the following were automatically generated by the web application.

**Table 3:** Development of a test for ER status with at least 90% sensitivity and 90% specificity.

Variable	GSE2034(training set)	GSE7390(validation set)
Reliable test results	93.4% (89.9%–95.7%)	84.3%
Sensitivity	90.4% (85.7%–93.7%)	91.8%
Specificity	90.9% (82.4%–95.5%)	87.5%

ER expression (reporter 205225_at) was the test variable that was compared to immunohistology that served as gold standard. In GSE2034 (training data set), one cutoff point was determined in such a way that the sensitivity exceeded 90% and another cutoff point was determined in such a way that the specificity exceeded 90%. Samples with expression between were considered to be equivocal with respect to the test result and excluded. The samples that are not in the equivocal zone between the cutoffs are considered as delivering a reliable test result. Sensitivity and specificity of the test are estimated based on these samples. A validation of the cutoffs 10.22 and 11.49 (determined in GSE2034) was executed in GSE7390. Percentages for the training set are reported including 95% confidence intervals.

### Fit of a Mixture Model

This method for cutoff determination is convenient for a molecular variable with bimodal shaped distribution. The cutoff point is optimized based on the hypothesis that the variable is distributed according to a mixture of two Gaussian distributions. Using this method, the histograms ER and PgR expression were generated and optimal cutoff points were determined (Fig. 2). Both distributions had a pronounced bimodal shape with cutoffs points located at 10.6 (ER) and at 5.0 (PgR).

### Optimizing the Correlation with Outcome

One of the most straightforward methods for cutoff optimization concerning a binary outcome variable is to maximize the significance of the 2×2 table. Other methods are discussed in a paragraph below. As example, we searched for a cutoff point for ER expression that optimally classifies with respect to ER status. In this analysis, ER status is determined by immunohistochemistry (IHC), the gold standard for investigation of ER and PgR. Fig. 3A shows the correlation of gene expression and ER status according to the designated cutoff point (Fig. 3A). The correlation was significant for almost all (99.6%) of the investigated cutoff points. The optimal cutoff was determined as 10.1 with odds ratio OR = 67.8 (30.2–152.1).

### Optimizing the Correlation with Survival

The straightforward method for determination of a prognostic cutoff point is to optimize the significance of the split in the Kaplan-Meier plot. As an example, we analyzed the correlation of gene expression determined PgR status and distance metastasis-free survival according to the designated cutoff point (Fig. 3B). The correlation was significant over a large range (70%) of the investigated cutoff points. The optimal cutoff was determined as 2.5 with hazard ratio HR = 0.46 (0.30–0.71). Additionally, a plot showing the difference in survival times between the tumors of high PgR expression and low PgR expression can be generated (Fig. 4). These results are in accordance with the strong prognostic relevance of PgR status for distance metastasis-free und overall survival in breast cancer [23], [24].

### Plots at Cutoff Point

Next, we investigated the dichotomization induced by the IHC-data-based and the survival-data-based cutoff determination in more detail. Using ERS1 expression measured by the microarray, determination of ER status was feasible with a sensitivity of 85.7% and a specificity of 91.9% (Fig. 5A). Kaplan Meier analysis showed a significantly better outcome for patients with high PgR expression (Fig. 5B, p = 0.00028).

### Comparison of Different Methods for Cutoff Optimization

For cutoff determination in the ER expression data, we compared the mixture model approach and three approaches based on comparison with immunohistologically determined ER status (Tab. 1). The latter approaches included optimization of the significance and of the distance of a point on the ROC curve from the upper left edge of the ROC diagram. One of the ROC based methods minimizes the Euclidean distance to this point, while the other minimizes the Manhattan distance to this point. The cutoff points determined by the significance method and by the Euclidean distance method were exactly the same for all data sets, while the Manhattan distance method agreed with these for one of the data sets and derived by only 0.3 for the other data set. The mixture model method showed a larger deviation from these results and between the three data sets. However, all cutoffs were between 9.6 and 11.2 resulting sensitivities greater than 88% and specificities greater than 81%.

For cutoff determination in the PgR expression data, we compared the mixture model approach and the approach based on the significance of the split in the Kaplan-Meier plot. The distribution based cutoffs for PgR expression were between 4.2 and 5.6 (Tab. 2). Interestingly, survival-data based cutoff determinate led to a reduction of the cutoff value in some data sets (GSE2034 and GSE11121) and to an increase of the cutoff value in another data set (GSE7390).

### Use Case: Development of a Molecular Test with Sensitivity and Specificity Greater than 90%

Finally, we discuss the development of a test for ER status from ER gene expression measured by the microarray probe 205552_at (Tab. 3). Using immunohistochemistry as gold standard and GSE2034 as training set, we executed two runs of Cutoff Finder to determine cutoff points with sensitivity >90% and with specificity >90%. Based on that, we defined a test result as positive, if it was above the upper threshold (11.49) and as negative, if it was below the lower threshold (10.22). The interval between the lower threshold and the upper threshold was defined as equivocal zone that contained only 6.6% of the samples in the training set. Using GSE7390 as validation set, test sensitivity was again above 90%, while test specificity was slightly below 90%, but inside the confidence interval estimated from the training set.

## Discussion

*Cutoff Finder* is a freely available in the internet (http://molpath.charite.de/cutoff). The web application is straightforward to use: The user can upload a molecular data set that optionally includes outcome and/or survival data. Then, the user selects the variables for analysis, the method for cutoff determination and the set of plots. On a result web page, the optimal cutoff point, overview plots and plots at the optimal cutoff point are displayed. The plots can be downloaded and included in scientific publications.

The problem of the choosing the optimal cutoff is difficult to answer generally. The best method for cutoff determination may depend on the biomarker, the assay and the clinical application under investigation. We exemplified this situation by analyzing the agreement between RNA based with immunohistology based determination of estrogen receptor status using different methods. It turned out that the cutoff points determined by three optimization methods in two data sets were very similar (all between 10.1 and 10.7). We believe that this is a consequence of the high concordance between gene expression and immunohistology in this use case, while in other situations cutoffs determined by different methods can differ substantially [6]. To cope with different biomarkers and assays, *Cutoff Finder* offers a multitude of five methods for cutoff optimization.

Stratification into two groups, but not into three or more groups, seems to be most natural for translation into clinics where most of the decisions are binary (treatment or no treatment). However, some of the gene tests for breast cancer stratify patients into a low risk and a high group (Mammaprint [25] and Endopredict [26]), while other additionally introduce a medium risk group (Oncotype DX [27]). An interesting feature of the software X-Tile is simultaneous optimization of two cutoffs leading to the identification of high, medium and low risk populations using a minimal p-value approach. X-Tile is a bioinformatic tool for prognostic cutoff optimization that is freely available to academic users [7]. *Cutoff Finder* is primarily specialized to the dichotomization situation. However, we provided an example of stratification into three groups by two subsequent runs: Patients were divided into a group where the test is highly sensitive, a group where the test is highly specific and a group of the remaining patients with equivocal test results.

Many of the methods for cutoff determination are based on optimization of a target quantity in dependence of the cutoff. For example, the target quantity can be the test accuracy or the significance of correlation of the biomarker with outcome or survival. It is clear that in such situation a multiple testing problem occurs that can lead to overestimation of the significance of the optimal cutoff and the effect size at the optimal cutoff [11], [12], [28]. Correction of p-values and confidence intervals for maximally selected chi square statistics and maximal selected log rank test was investigates in the 1980s and the 1990s [29]–[32]. *Cutoff Finder* addresses the multiple testing problem by visualizing odds ratios or hazard rations including confidence intervals for each of the cutoffs under investigation. This kind of plots together with the proportion of significant tests can help to assess the stability and significance of the dichotomization. Further, we recommend integrating cutoff optimization in a sequential biomarker study design: In the first step of such an approach, the cutoff is optimized in one or more retrospective studies. In the second step, the predefined cutoff is validated in one or more retrospective or prospective studies. Both steps can be executed using *Cutoff Finder*.

Pathologists often use packages like SPSS (SPSS, Inc., an IBM Company), GraphPad Prism (GraphPad Software, Inc.) or Winstat (R.Fitch Software) in order to correlate biomarkers with outcome or survival data. However, neither of them includes functions for cutoff optimization. Therefore, cutoffs are often chosen as median or mean value of the biomarker or adjusted manually. For example, percentile based cutoffs were also implemented in our previous project where biomarkers can be assessed for their association to survival [33]. The commercial software JMP (SAS, Inc.) allows for optimizing a dichotomization for correlation with a binary outcome variable in a multivariate context. However, distribution-based cutoff optimization or cutoff optimization in context of a survival variable is not supported. The STEPP method allows graphical assessment of treatment-covariate interactions using Cox models on subsets of the data [34]–[36]. However, STEPP addresses the analysis of a clinical study in a series of overlapping patient subgroups rather than optimization of a cutoff point.

A specialty of Cutoff Finder is the plot of ORs and HRs including confidence intervals. The plots include information on both, the strength and significance of the prognostic stratification. This is in particular important in complex situations where ORs or HRs change strongly in dependence of the cutoff. For example, in a study on Ki67 protein expression in lung cancer, we observed positive HRs for low cutoffs and negative HRs for high cutoffs (data not shown).

*Cutoff Finder* was developed to facilitate cutoff optimization for tissue biomarkers that are investigated in pathology research. Additionally, we expect our software to be interesting for a wider group of scientists, including clinical chemists and other biomedical investigators, who want to convert a metric or ordinal variable into a dichotomization. As an example, we analyzed the expression of hormone receptors in breast cancer tissues. Cutoff point determination using different analysis modes turned out to be feasible and useful. In summary, we presented a comprehensive and straightforward tool for cutoff point optimization that can help to improve the quality of biomarker studies.

## Supporting Information

Text S1 **R code of the functions that are used for cutoff optimization and the generation of plots.**
(R) Click here for additional data file.
